# A supramolecular protein chaperone for vaccine delivery

**DOI:** 10.7150/thno.39132

**Published:** 2020-01-01

**Authors:** Zhongyan Wang, Yuna Shang, Zhaoqi Tan, Xiaoyan Li, Guoliang Li, Chunhua Ren, Fuqiang Wang, Zhimou Yang, Jianfeng Liu

**Affiliations:** 1 Tianjin Key Laboratory of Radiation Medicine and Molecular Nuclear Medicine, Institute of Radiation Medicine, Chinese Academy of Medical Sciences & Peking Union Medical College, Tianjin 300192, P.R. China.; 2 Key Laboratory of Bioactive Materials, Ministry of Education, College of Life Sciences, State Key Laboratory of Medicinal Chemical Biology, Collaborative Innovation Center of Chemical Science and Engineering, and National Institute of Functional Materials, Nankai University, Tianjin 300071, P. R. China.; 3 Analysis Center, Nanjing Medical University, Nanjing, Jiangsu 210029, P. R. China.; 4 Lab of Functional and Biomedical Nanomaterials, College of Materials Science and Engineering, Qingdao University of Science and Technology, Qingdao, 266042, P. R. China.; 5 Jiangsu Center for the Collaboration and Innovation of Cancer Biotherapy, Cancer Institute, Xuzhou Medical University, Xuzhou, Jiangsu, P. R. China.

**Keywords:** Self-assembly, peptide, protein delivery, vaccine adjuvant, immunotherapy

## Abstract

**Rationale**: Nanomaterials capable of specifically interacting with proteins are very important for protein storage and vaccine delivery. Supramolecular hydrogels based on peptides have emerged as promising vaccine adjuvants because of their good compatibility, ease of antigen incorporation and display, and efficiency in activating immune responses.

**Methods**: We synthesized a self-assembling peptide (Fbp-G^D^F^D^F^D^Y^D^K(γE)_2_-NH_2_, ***Comp. 1***) serving as a supramolecular protein chaperone for protein antigen delivery. The gelation was triggered by simply mixing ***Comp. 1***and proteins. The vaccine adjuvant potential of ***Comp. 1*** was demonstrated by using two protein antigens, ovalbumin (OVA) and hepatitis B surface antigen (HBsAg).

**Results**: The peptide derivative ***Comp. 1***exhibited high protein binding capacity. Upon contacting proteins, ***Comp. 1***rapidly formed coassembled nanofibers/hydrogels with the proteins, which greatly delayed the release of protein antigens. Our supramolecular protein chaperone significantly stimulated specific antibody titers by assisting protein delivery to antigen-presenting cells, promoting dendritic cell (DC) maturation, prolonging antigen accumulation and retention in the lymph nodes, and eliciting the secretion of cytokines. Most importantly, our supramolecular protein chaperone strongly stimulated the cellular immune response and significantly retarded tumor growth.

**Conclusion**: Our study demonstrated the great potential of the supramolecular protein chaperone in protein storage and delivery, vaccine production and tumor immunotherapy.

## Introduction

Tumor immunotherapy, including immune checkpoint blockade therapy, immune cell therapy and tumor vaccine therapy, has received increasing attention in recent years 1-3. As an important part of tumor immunotherapy, therapeutic tumor vaccines have recently attracted extensive attention. In preclinical or clinical trials, tumor vaccines can induce innate and durable adaptive immunity. Many clinical trials have exhibited that tumor vaccines have good tolerance and safety while obtaining antigen cascade reaction 4. The combination of tumor vaccines and other tumor therapies like surgery, chemotherapy and radiotherapy, especially immunocheckpoint inhibitors, may lead better clinical therapeutic effect as well as reduce the adverse reactions 5. The identification of tumor cell-specific antigens and the development of CD8^+^ T cell-stimulating vaccine adjuvants are two important steps in the development of successful tumor vaccines 6-8. High-throughput screening and gene microarrays have been demonstrated to be powerful techniques for identifying tumor cell-specific peptide antigens, which has led to the development of personalized vaccines to treat melanoma 9,10. However, limited vaccine adjuvants hinder the development of therapeutic cancer vaccines. Nanomaterials based on organic materials (e.g., poly(lactic-co-glycolic acid) (PLGA)/polylactic acid (PLA) nanoparticles and polymeric hydrogel) and inorganic materials (e.g., gold nanostructures and silica nanomaterials) have been demonstrated to be promising vaccine adjuvants that promote innate and adaptive immune responses 11-16. The formulation of vaccines using these adjuvants is complicated and sometimes not suitable for protein or peptide antigens. Recently, in addition to their important applications in nanomedicine 17-24, tissue engineering 25-27 and bioanalysis 28-31, hydrogels of self-assembling peptides have emerged as promising vaccine adjuvants because of their good compatibility, ease of antigen incorporation and display, and efficiency in activating immune responses 32-36. Molecules or nanomaterials capable of interacting with protein antigens and coassembling with them are very useful for protein antigen storage and vaccine delivery, which can improve the immunogenicity of protein antigens by preventing them from rapid degradation 37. However, there are few successful examples being developed by the specific peptide-protein or aptamer-protein interactions 38,39. In this study, we introduced a supramolecular protein chaperone capable of forming coassembled nanofibers/hydrogels by binding protein antigens for the development of promising vaccines including therapeutic cancer vaccines and hepatitis B vaccines.

## Materials and Methods

### Materials

Rink Amide Resin (1.2 mmol/g) and 2-Cl-trityl chloride resin (1.1 mmol/g) were obtained from Nankai University resin Co., Ltd. Fmoc-amino acids and o-benzotriazol-1-yl-N,N,N′,N′-tetramethyluronium hexafluorophosphate (HBTU) were received from GL Biochem (Shanghai). Chemical regents and solvents were used as received from commercial sources. All the other starting materials were obtained from Alfa. Rapamycin was purchased from Alladin. 3-methyl adenine was purchased from J&K scientific. Cyanine5.5 NHS ester was purchased from Seebio. Horseradish peroxidase-conjugated goat antimouse IgG, IgG1, IgG2a, or IgG2b were obtained from Southern Biotechnologies (AL, USA). Recombinant mouse GM-CSF and IL-4 were purchased from Peprotech (Rocky Hill, USA). Mouse IL-5, IFN-γ, TNF-α, IL-2, IL-6, and IL-12 ELISA kits were obtained from Biolegend (CA, USA). Fluorochromelabeled anti-mouse monoclonal antibodies (PE-CD40, APC-CD80) and endotoxin free ovalbumin (OVA, endotoxins <1 EU/mg) were purchased from InvivoGen (CA, USA). Alum adjuvant was purchased from Pierce Biotechnology (IL, USA). Goat anti-mouse α-SMA, CD206, CD3 and iNOS antibodies were purchased from Abcam. Six to eight-week-old C57BL/6 mice were purchased from Beijing Vital River Laboratory Animal Technology Co., Ltd. and maintained in specific pathogen-free conditions in the animal facility at the Nankai University, Tianjin, China.

### Synthesis and characterization of peptides

Peptides were synthesized by standard peptide solid phase synthesis (SPPS) method. The Rink amino resin first was used to synthesize Fbp-G^D^F^D^F^D^Y^D^K. The side chain of lysine was protected with 4-methyl trityl (Mtt), 1% trifluoroacetic acid (TFA) can take off Mtt group without removing peptide from the resin. Then Fmoc-Glu-OtBu and Boc-Glu-OtBu were conjugated to lysine side chain amino. The peptide Fbp-G^D^F^D^F^D^Y^D^K(γE)_2_-NH_2_ finally was cut off from the resin with 95% TFA. The crude peptide was separated and purified by reversed-phase high performance liquid chromatography (HPLC), and the structure of the compound was characterized by high resolution mass spectrometry and nuclear magnetic hydrogen spectrometry.

### Preparation of hydrogel

2.5 mg of compounds were dispersed in endotoxin-free PBS buffer solution (pH = 7.4) at a final concentration of 0.5 wt% with sodium carbonate to adjust the final pH to 7.4. Proteins were dispersed in endotoxin-free PBS buffer solution (pH = 7.4) at a final concentration of 10 μg/μL. The proteins were then evenly dispersed in the hydrogel by vortexing. The mixtures were maintained at 37°C for half hour and hydrogel then formed. The final concentration of the protein in the hydrogel was 500 μg/mL.

### Co-assembly certification

The fluorescence binding assay was performed on a total internal reflection fluorescence microscope (TIRFM) imaging system (Nikon Ti-U inverted epi-fluorescence microscope). Rhodamine (λ*_exc._* = 554 nm)-labelled OVA was used to induce hydrogelation and (E)-4-(2-(9-(2-(2-methoxyethoxy)ethyl)-9H-carbazol-3-yl)vinyl)-1-methylquinolinium iodide (SLM) (λ*_exc._* = 488 nm) was used to label the Aβ peptides and fibrils. The fibrils were immobilized on a standard glass coverslip functionalized with amino groups. The un-fixed fibrils and other molecules were washed away with deionized water. The fluorescence from the dye was collected by a 100×TIRF objective (NA 1.49) and then recorded with an Andor iXon 897 EMCCD.

### Microscale thermophoresis

Proteins were labeled with the fluorescent dye NT-647 using Monolith NT™ Protein Labelling Kits (cysteine-reactive) (NanoTemper Technologies, Germany). PBS buffer containing 0.05% Tween-20 (pH = 7.4) was used as the assay buffer. For the interaction experiments of fluorescent-proteins with ***Comp. 1*** or ***2***, the concentration of labelled proteins were maintained constant, while the concentration of ***Comp. 1*** or ***2*** varied from 0.25 μM to 10 mM. Then the solution of fluorescent-proteins was mixed with solutions containing different concentrations of ***Comp. 1*** or ***2*** at 1:1 volume ratio. After a short incubation time, the samples were loaded into MST NT.115 standard glass capillaries and the analysis was performed using the Monolith NT.115 system (NanoTemper Technologies, Germany). The K_D_ value was calculated using the NanoTemper software package.

### Antigen release assay

The released profiles of OVA-RBITC from Vac-1 and Vac-2 *in vitro* were studied at 37 °C. 150 μL of Vac-1 or Vac-2 (0.2 wt%) containing 30 μg of OVA-RBITC was used for the measurement. 150 μL PBS solution (pH = 7.4) was firstly added on top of the gel, 100 μL of solution was taken out at the desired time point and 100 μL of fresh PBS solutions was added back. The absorbance of OVA-RBITC was determined at 560 nm by a microplate reader (BioTek Synergy 4) to calculate the cumulative release rate of OVA-RBITC from hydrogel vaccines.

### Analysis of *in vivo* stability of hydrogel vaccines

Cy5.5-G^D^F^D^F^D^Y^D^K(γE)_2_-NH_2_ and Cy5.5-G^D^F^D^F^D^Y were synthesized by SPPS (Cyanine5.5 NHS ester as a replacement of flurbiprofen). OVA was evenly mixed into the solution of Cy5.5-G^D^F^D^F^D^Y^D^K(γE)_2_-NH_2_ at a concentration of 0.2 wt% for self-assembly. C57BL/6 mice were subcutaneously administered with a final volume of 100 µL vaccines in inguinal region. The fluorescence images were recorded every 6 or 12 hours at 640 nm excitation wavelength by Cri Maestro In-vivo imaging System (Xenogen, IVIS Lumina II).

### Evaluation of immune efficacy of vaccines

*In vivo* immune evaluation, female C57BL/6 mice were randomly divided into four groups and each group contains five mice. Every mouse was subcutaneously administered with a final volume of 100 µL vaccines (100 μL PBS with 20 μg OVA, 20 μg OVA with 25 times Alum and 0.2 wt% hydrogel vaccines composed of 20 μg OVA, respectively). The first and second immunizations were given at day 0 and 14. Day 7 after the second immunization, serum was collected for the antibody detection and splenocytes were collected for the production of cytokine assay. OVA-specific antibody responses in mice were examined by using ELISA. 96-well ELISA plates were coated with 10 μg/mL OVA antigen and stored at 4 °C overnight. After three washes with PBST (PBS buffer containing 0.05% Tween-20), the plates were blocked by using blocking buffer (1% BSA in PBST solution) for 1 h at room temperature. Individual antisera were serially diluted in the blocking buffer and incubated in the wells for 2 h. After five washes with PBST, the wells were incubated with goat anti-mouse IgG horseradish peroxidase for 1 h. After washing 5 times, antibody binding was assessed by adding 100 μL of the 3,3′,5,5′-tetramethylbenzidine peroxidase substrate to each well. The substrate reaction was terminated by adding 50 μL of 2 M H_2_SO_4_. Antibody isotypes were determined similarly using goat anti-mouse IgG1, IgG2a and IgG2b horseradish peroxidase. The plates were then read by using an ELISA reader at an optical density of 450 nm. Antibody titers were calculated as the reciprocal serum dilution giving O.D. readings > 0.1 standard deviations above the background levels as calculated using PBS at the same dilutions.

### The effect of vaccines on splenocytes proliferation

At 7 days after the second immunization, splenocytes were labeled with CFSE, and then treated with soluble OVA (50 μg/mL), BSA (50 μg/mL), or Medium at 37 ºC for 72 h. The splenocytes were harvested and analyzed by flow cytometry. At the same time, splenocytes (5×10^6^ cells/mL) were seeded in 24-well plates, and then were retreated with soluble OVA (50 μg/mL) at 37 ºC for 96 h. The production of IFN-γ and IL-5 in cell supernatants were detected by using ELISA kit.

### The effect of vaccines on promotion of dendritic cells maturation

Bone marrow cells were isolated from C57BL/6 mouse femur and tibia, and then cultured in X-vivo 15 medium (Lanza, MD, USA) containing GM-CSF (20 ng/mL) and IL-4 (10 ng/mL) at 37 °C for 6 days to acquire immature DCs. After 7 days, the immature DCs were collected and washed with PBS buffer for 3 times. 250 µL Vac-1 (2 mg/mL) or Vac-2 (2 mg/mL) was evenly mixed into 10 mL fresh X-vivo medium by vortex for stimulating DCs. The immature DCs were seeded in 6-well plate and incubated with 2 mL X-vivo medium containing Vac-1 or Vac-2. The plate was put in a cell incubator (ThemoFisher Scientific) at 37 °C for 24 h. At the end, the DCs were harvested and incubated with appropriately diluted PE-antimouse-CD40 and APC-antimouse-CD80 monoclonal antibodies on ice for 30 minutes. The expressions of CD40 and CD80 on BMDCs were detected by flow cytometry. Cytokine production was detected by using ELISA kits.

### Preparation of OVA-Rhod and evaluation of enrichment in lymph nodes

5 mg OVA and 2 equivalent of Rhodamine b isothiocyanate were dissolved in 1 mL of PBS and added 1 equivalent Na_2_CO_3_ to adjust the final pH to 7.4. The mixture was put into a dialysis bag (interception molecular weight: 1000 Da) and stirred at 4°C for 24 hours. The protein concentration after dialysis was determined by NanoDrop analyzer. The OVA-Rhod was mixed with PBS, Alum, ***Comp. 1*** and ***Comp. 2*** (100 μL PBS, 20 μg OVA with 100 μL PBS, 20 μg OVA with 25 times Alum, 20 μg OVA with 200 μg compounds, per mouse respectively). The mice were randomly injected with 100 μL mixtures per mouse, and lymph nodes at the proximal and distal ends were taken 24 hours and 48 hours later, respectively. Then, the fluorescence intensity was measured by a live animal imager (exposure time: 4s).

### The influence of vaccines on macrophages autophagy

To acquire Bone marrow-derived macrophages (BMDMs), bone marrow cells isolated from C57BL/6 mouse femur and tibia firstly were treatment with ACK lysis buffer, and then were cultured in Dulbecco's modified Eagle medium containing macrophage colony-stimulating factor (M-CSF, 10 ng/mL), 10% FBS, 1% P/S and 2 mM glutamax at 37 °C for 7 days. Subsequently, the BMDMs were stimulated with 50 µL Vac-1 (2 mg/mL) or Vac-2 (2 mg/mL) for 24 h. Rapamycin (Rap, Aladdin) or 3-methyl adenine (3MA, J&K scientific) was used to promote or inhibit autophagy, respectively. LC3 puncta images of autophagosome in BMDMs stained with LC3-II antibody (FITC, Biorbyt) were detected by a confocal microscope (Leica TCS SP5). IL-2 cytokine in BMDMs supernates was measured by ELISA kits (Biolegend).

### Evaluation of anti-tumor efficacy of vaccines

All mice were subcutaneously injected with E.G.7-OVA cells (5×10^5^ cells per mouse) on the right buttock, and when the tumor volume reached 100 mm^3^, they were randomly divided into four groups, five in each group. 100 μL PBS or hydrogels vaccines (including 20 μg OVA) were administered on day 6 and 13 for treatment per mouse. Tumor volume was measured every two days. Tumor volume = 0.5 × (length) × (width)^2^. The treatment of B16-OVA tumor was same as above.

### Histology assay

Tumor tissue from different groups were collected for immunohistochemistry analysis after sacrifice. After being fixed and dehydrated, all tumor samples were embedded in paraffin and cut into sections with a thickness of 5 μm. Immunohistochemistry staining was performed to determine specific markers. Tissue sections were firstly deparaffinized and rehydrated. Then, the antigens were retrieved from the tissue sections and the endogenous peroxidase were blocked with 3% H_2_O_2_. The cell nuclei were stained with hematoxylin solution. The sections were incubated with anti-α-SMA, anti-CD206, anti-iNOS or anti-CD3 overnight. The sections were washed with PBS and then incubated with secondary antibodies labeled with horseradish peroxidase (HRP)-conjugated goat anti-mouse IgG for 2 hours at 25 ^o^C. After washing each section with PBS for 3 times, the newly configured DAB chromotic solution was added by drops. The cell nuclei were stained with hematoxylin solution. The slides were observed under the microscope (Zeiss Axio Imager Z1, Germany).

### Statistical analysis

Statistical analysis was processed in GraphPad Prism 5. All data were shown as the mean ± standard error of mean (SEM), difference among groups are determined with analysis of variance (ANOVA) analysis. *p<0.05, **p<0.01 and ***p<0.001 were used to show statistical significance.

## Results and Discussion

### Molecular design and peptide synthesis

**Figure 1:**
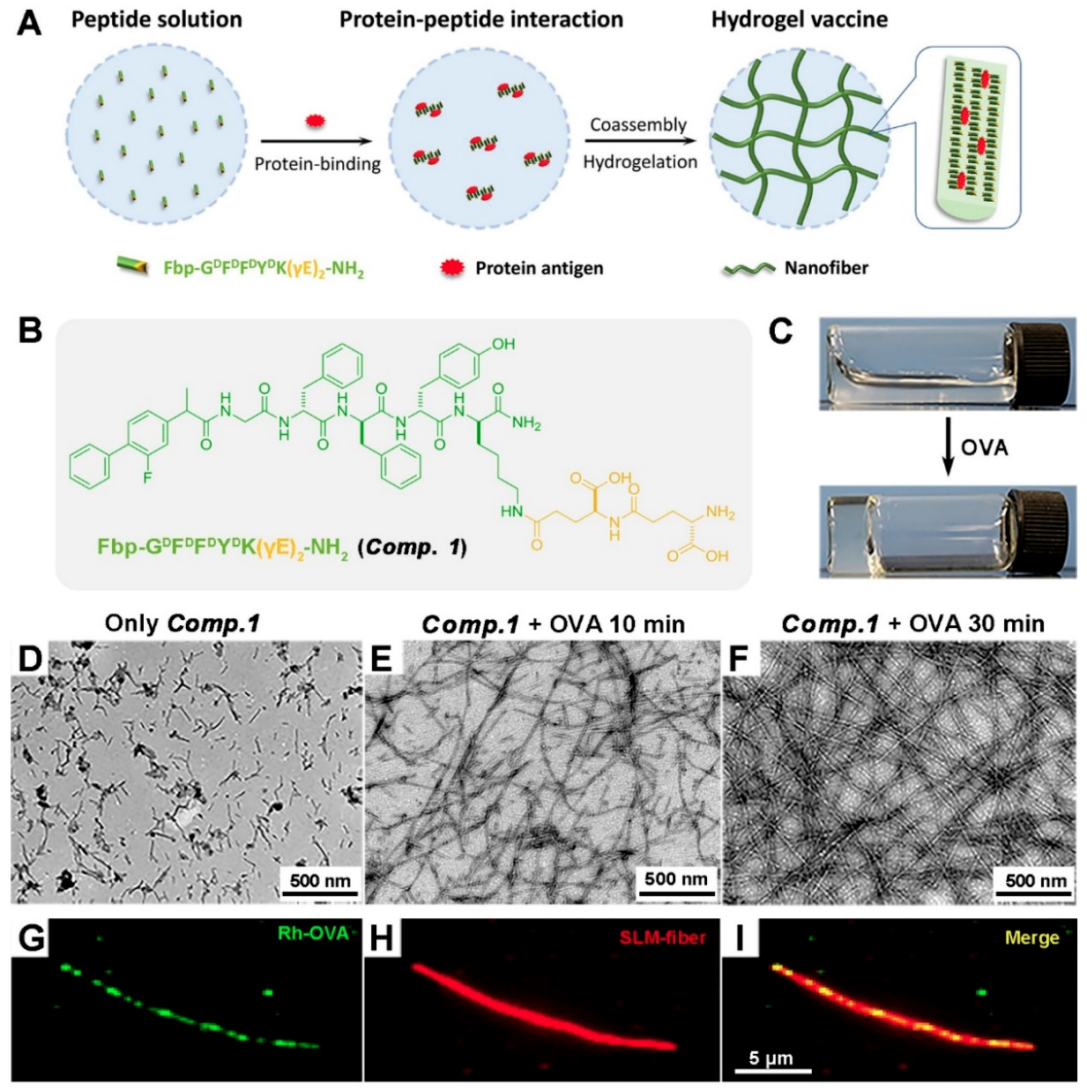
The course of supramolecular protein chaperone fibration/gelation. A) Schematic of the preparation of protein-induced coassembled hydrogel vaccine. (B) Chemical structure of ***Comp. 1***. (C) Optical images of a solution of ***Comp. 1*** (0.5 wt%) and the corresponding gel formed by adding OVA. (D-F) TEM images of a solution of ***Comp. 1*** and the gels formed at 10 minutes and 30 minutes after adding 10% OVA (0.05 wt%). (G-I) Total internal reflection fluorescence microscopy (TIRFM) images of the nanofibers in the gel containing ***Comp. 1*** and OVA.

We recently developed a supramolecular protein glue based on a peptide containing γ-glutamic acids (γEs) (Nap-GFFYK(γE)_2_-NH_2_), which was capable of specifically binding and coassembling with a variety of proteins to form supramolecular nanofibers and hydrogels 40. We intended to further explore its application in protein vaccine delivery, and we therefore designed the molecule Fbp-G^D^F^D^F^D^Y^D^K(γE)_2_-NH_2_ (***Comp. 1***, Figure 1B) and envisioned that ***Comp. 1***could coassemble with protein antigens to form coassembled nanofibers for efficient protein antigen delivery (Figure 1A). The flurbiprofen (Fbp; a nonsteroidal anti-inflammatory drug)-modified D-tetrapeptide Fbp-G^D^F^D^F^D^Y (***Comp. 2***) has been demonstrated to be a powerful tumor vaccine adjuvant 41. The peptide Fbp-G^D^F^D^F^D^Y^D^K(γE)_2_-NH_2_ was synthesized by standard Fmoc solid phase peptide synthesis (SPPS) using rink resin. Pure ***Comp. 1*** was obtained by high-performance liquid chromatography (HPLC).

### Preparation of protein antigen-induced hydrogel

We then tested the coassembly property of ***Comp. 1*** with the model protein antigen ovalbumin (OVA). ***Comp. 1*** dissolved well in phosphate-buffered saline (PBS; pH = 7.4) at a concentration of 0.5 wt%. After adding OVA to the solution (final protein concentration = 0.05 wt%), we observed rapid hydrogelation within 10 minutes (Figure 1C). The resulting hydrogel was stable for more than 3 months at room temperature (20-25 °C). The maximum proportion of the nanofibers composed of OVA was 50% for ***Comp. 1*** (Figure S5 and S6).

### Characterization of protein antigen-induced hydrogel vaccine

**Figure 2:**
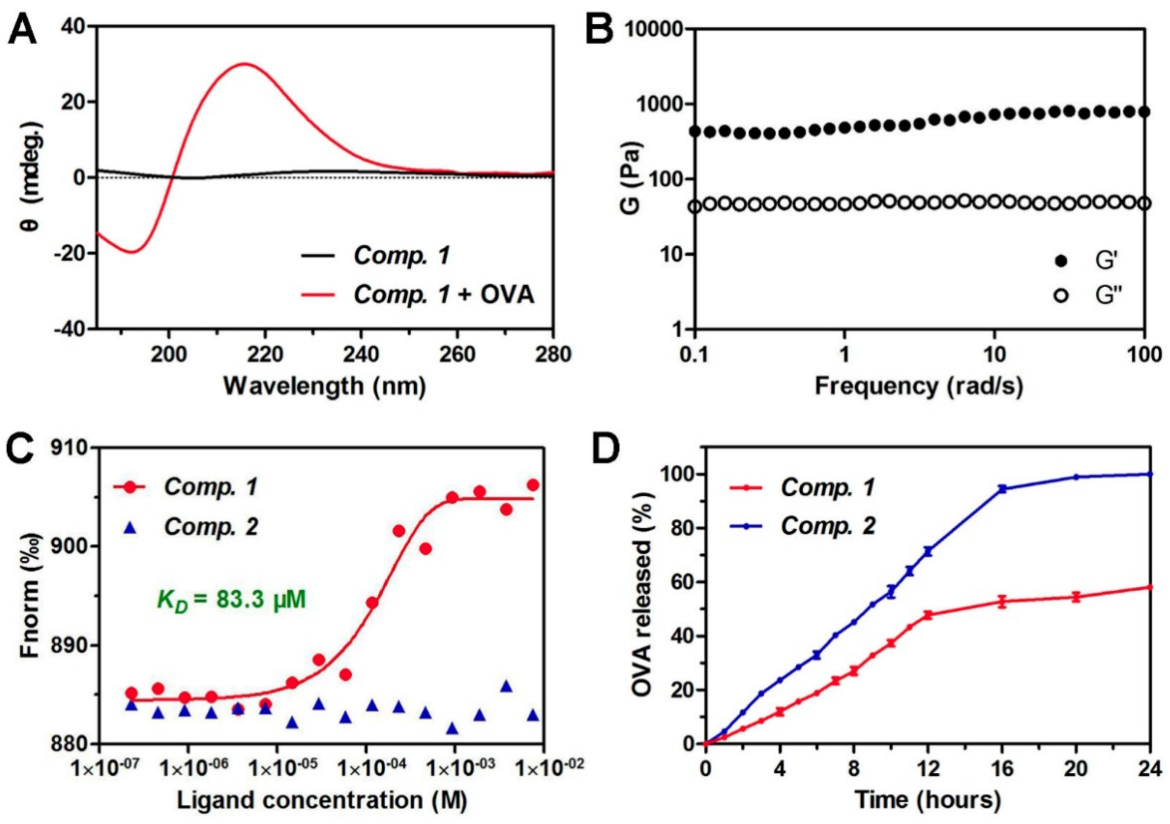
Characterization of protein antigen-induced hydrogel vaccine. (A) Circular dichroism (CD) spectra of hydrogels containing 0.5 wt% of ***Comp. 1*** and 0.05 wt% OVA. (B) Dynamic frequency sweep of hydrogel of ***Comp. 1*** containing 10 % amounts of OVA at strain of 0.1% at 37 ^o^C. (C) The fitting curve of microscale thermophoresis (MST) to calculate the K_D_ values between ***Comp. 1*** or ***Comp. 2***and OVA. (D) The profiles of OVA release from ***Comp. 1*** and ***Comp. 2*** at pH 7.4 at 37 ^o^C.

We then characterized the microstructure of the hydrogel by transmission electron microscopy (TEM). A PBS solution containing ***Comp. 1*** showed very short nanofibers with diameters of approximately 9.8 nm and lengths shorter than 200 nm (Figure 1D). Upon the addition of OVA, the short nanofibers gradually grew into longer nanofibers (Figure 1E). A three-dimensional network of uniform and long nanofibers with diameters of approximately 10.9 nm was observed at the 30 minute time point (Figure 1F). During the peptide self-assembly, the OVA served as coacervation stimulator may facilitate peptide nucleation and growth of nanofibers 42. To validate the co-assembly between ***Comp. 1*** and OVA, we performed fluorescence colocalization assay via total internal reflection fluorescence microscopy (TIRFM). Rhodamine B-labeled OVA was used to induce hydrogelation of ***Comp. 1***. Then ***Comp. 1***in nanofibers were stained with (E)-4-(2-(9-(2-(2-methoxyethoxy)ethyl)-9H-carbazol-3-yl)vinyl)-1-methylquinolinium iodide (SLM) for TIRFM imaging 43. As shown in Fig. 1G-1I, the OVA and ***Comp. 1***colocalized in nanofibers, convincingly confirming that the nanofibers in hydrogel were formed by supramolecular co-assembly of OVA and ***Comp. 1***. Similar to the supramolecular protein glue, the hydrogel was formed due to the induction of the β-sheet folding of ***Comp. 1*** by the addition of the protein OVA 40. As shown in Figure 2A, ***Comp. 1*** exhibited very weak circular dichroism (CD) signals but adopted a typical β-sheet secondary structure after the addition of 10 wt% OVA, as indicated by the negative peak at 192 nm and the positive peak at 215 nm. ***Comp. 1*** consisted of D-amino acids, and therefore, its CD pattern was the reverse of that of β-sheet L-peptides. We tested the mechanical property of coassembled hydrogel by a rheometer and found the hydrogel had moderate and stable property, which was conducive to injection and sustained release (Figure 2B). We also performed a microscale thermophoresis (MST) assay to determine the binding affinity (K_D_) between ***Comp. 1*** and OVA. As shown in Figure 2C, ***Comp. 1***exhibited a good binding affinity for OVA with a K_D_ value of 83.3 μM, while Fbp-G^D^F^D^F^D^Y (***Comp. 2***) had no measurable binding affinity for OVA. The protein OVA was released gradually from the coassembled nanofibers. As shown in Figure 2D, approximately 60% of the coassembled OVA protein was released within 24 hours. However, the physically encapsulated OVA in the hydrogel created with ***Comp. 2*** was released rapidly, and nearly 100% of the OVA protein was released within 16 hours. According to the results, the high binding affinity of ***Comp. 1***and OVA is the trigger for changing the hydrophilicity of peptide, and the non-covalent bond (e.g. hydrogen bond, π-π interaction) among peptide molecules is the basis of self-assembly, resulting in formation of nanostructure with unified conformation. Meanwhile peptide-binding OVA was also loaded into the hydrogel. The hydrogelation strategy suggested that ***Comp. 1***has great potential in protein antigen storage and delivery.

### Biocompatibility and stability *in vivo*

A biocompatibility assay indicated that 80 µg/mL ***Comp. 1*** or ***Comp. 2*** did not remarkably reduce the cell viability of either splenocytes or Raw 264.7 mouse macrophages (Figure S7). To explore the stability of our hydrogel vaccines *in vivo*, we replaced flurbiprofen with Cyanine5.5 NHS ester (Cy5.5, λ*_exc._* = 640 nm) to synthesize Cy5.5-G^D^F^D^F^D^Y^D^K(γE)_2_-NH_2_ and Cy5.5-G^D^F^D^F^D^Y for imaging *in vivo*. The modified vaccines (Vac-1_m_ or Vac-2_m_) were injected to inguinal region of C57BL/6 mice and the fluorescence signal was detected by living animal imaging system. Our protein chaperone show longer retention time of about 48 h in injection site, while Vac-2 only remained there for about 24 h, further indicating that our protein chaperone has stable mechanical properties and ability of anti-enzyme degradation (Figure S8). The good biocompatibility and stability *in vivo* of Vac-1 suggested it has great potential in vaccine development.

### Vaccine enhancing production of specific antibodies and cytokines

**Figure 3:**
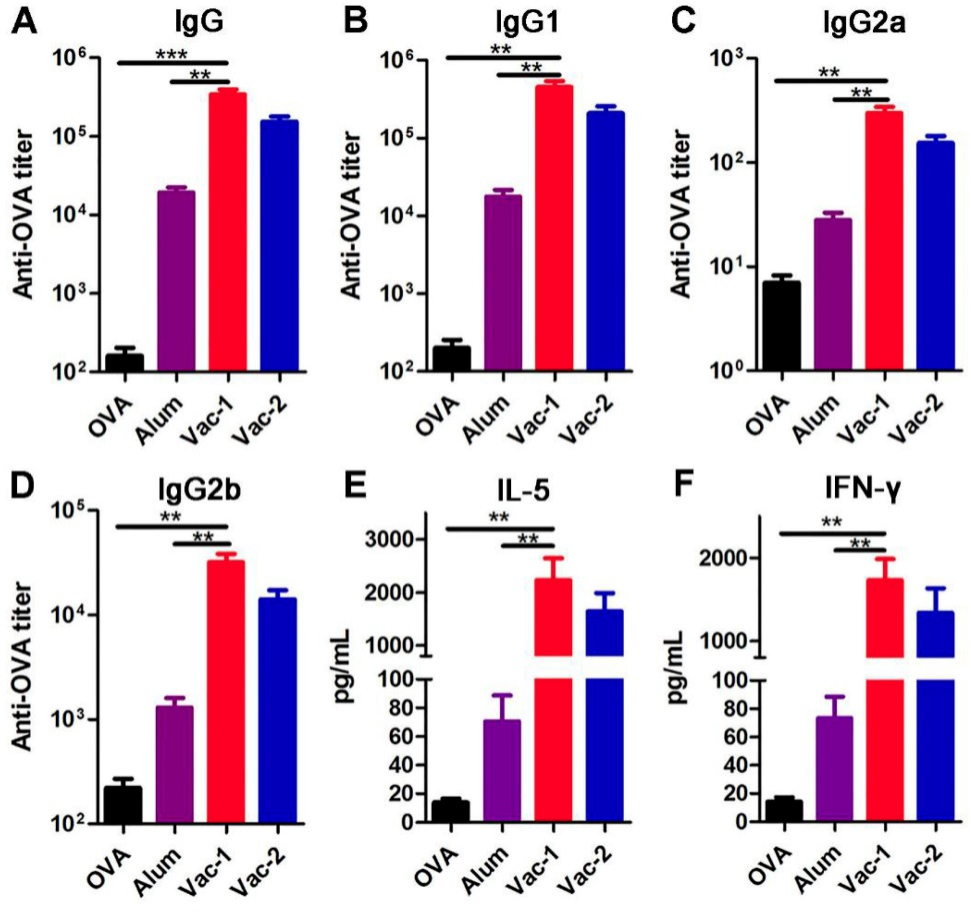
Determination of OVA-specific antibody titers and splenocyte cytokines. The levels of anti-OVA (A) IgG, (B) IgG1, (C) IgG2a and (D) IgG2b antibodies in plasma on day 21 were detected using ELISA. The secretion of (E) IL-5 and (F) IFN-γ into supernatants was detected using ELISA. The bars represent the mean ± SE, and differences among groups were determined using one-way ANOVA. The asterisks indicate the significance of differences between the Vac-1 group and the indicated group. *: *p* < 0.05, **: p < 0.01, and ***: p < 0. 001.

We then prepared two hydrogel vaccines and tested their abilities to stimulate antibody production. The hydrogel containing ***Comp. 1*** (0.2 wt%) and OVA (10 wt% to ***Comp. 1***) was prepared in a 37 °C incubator for half an hour (Vac-1). The hydrogel containing ***Comp. 2*** was formed by heating and cooling was mixed with 10 wt% OVA with a vortex (Vac-2). Both hydrogel vaccines were subcutaneously administered twice (on day 7 and day 14), and then antibody titers were measured by ELISA on day 21. Compared with the Alum adjuvant, both Vac-1 and Vac-2 significantly increased the IgG antibody titer by 17.7-fold and 8-fold, respectively (Figure 3A). The titer of IgG antibody against OVA elicited by Vac-1 was 2.22-fold higher than that elicited by Vac-2, suggesting that the coassembly strategy was more efficacious than simple physical encapsulation for antibody production. With regard to IgG subtypes, compared to the Alum adjuvant, Vac-2 immensely enhanced the production of anti-OVA IgG1, IgG2a and IgG2b antibodies (Figure 3B-3D), which was consistent with our previous results. However, compared with Vac-2, Vac-1 tremendously promoted the secretion of anti-OVA IgG1, IgG2a and IgG2b antibodies, with increases of approximately 2-fold (Figure 3B-3D). Cytokines secreted by T lymphocytes play important roles in immunoregulation. Th2 cell cytokines (e.g., IL-5) can promote proliferation and differentiation in B cells, which helps the production of specific antibodies (e.g., IgG1 antibodies). On the other hand, Th1 cell cytokines (e.g., IFN-γ) can regulate Ig isotype switching and promote IgG2a and IgG2b antibody production as well as the differentiation and cytotoxicity of cytotoxic T lymphocytes (CTLs). As shown in Figure 3E and 3F, Vac-1 more pronouncedly increased the production of OVA-specific IFN-γ than did Vac-2, which was consistent with their effects on IgG2a and IgG2b antibody production. In addition, Vac-1 more potently enhanced the production of IL-5 than did Vac-2 or Alum, which was consistent with Vac-1 eliciting the highest IgG1 titer among all treatments (Figure 3E). Furthermore, compared to other vaccines, Vac-1 promoted the proliferation of OVA-specific splenocytes by 1~3 fold (Figure S9). No difference was observed among the groups once the protein antigen was replaced with an unrelated protein, indicating that splenocyte proliferation was highly antigen dependent.

**Figure 4:**
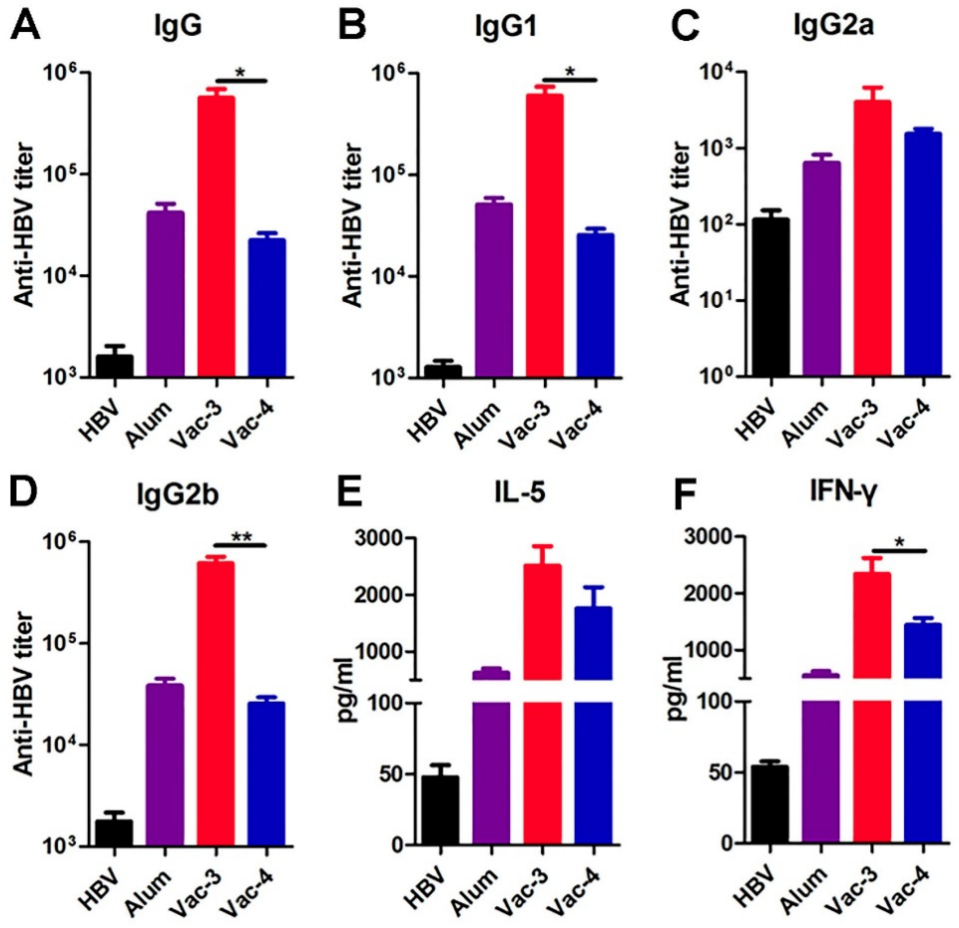
Determination of HBsAg-specific antibody titers and splenocyte cytokines. (A-D) The production of anti-HBsAg IgG (A), IgG1(B), IgG2a (C) and IgG2b (D) antibodies in plasma on day 21 were detected using ELISA. The secretion of IL-5 (E) and IFN-γ (F) in the splenocyte supernatants were detected using ELISA.

To confirm whether our antigen-chaperone peptide could also be utilized with other antigens, we measured its efficacy as a hepatitis B virus (HBV) vaccine adjuvant. Hepatitis B surface antigen (HBsAg) is a coat protein of HBV and has a binding affinity of 68.05 μM for ***Comp. 1***(Figure S10). Serum was collected from C57BL/6 mice one week after a second immunization to measure anti-HBsAg antibody titers using ELISA. As shown in Figure 4, the antibody titers and cytokines elicited by the vaccine made from ***Comp. 1*** were higher than those elicited by other treatments, suggesting the substantial potential of our supramolecular protein chaperone for the development of vaccines to prevent infectious diseases.

### Vaccine promoting antigen uptake and dendritic cells maturation

**Figure 5:**
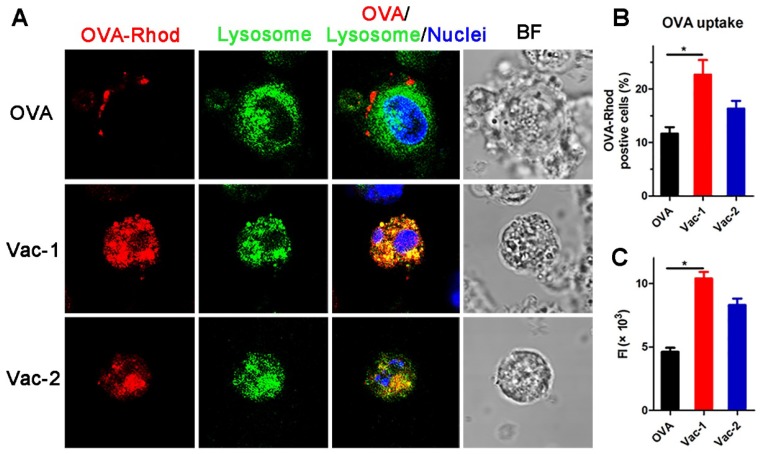
Vaccine promoting antigen uptake by BMDCs. (A) BMDCs were incubated with soluble OVA-Rhod (2.5 µg mL^-1^), Vac-1 (***Comp. 1***/OVA = 10:1, w/w), or Vac-2 (***Comp. 2***/OVA = 10:1, w/w) at 37 °C for 1 h, stained with LysoTracker and Hoechst dye, and evaluated by confocal laser scanning microscopy. Magnification: 63 ×. (B) The percentage of OVA-Rhod-positive cells is shown. (C) Total fluorescence intensity of OVA-Rhod was measured; total fluorescence intensity (FI) = % of positive cells × mean fluorescence intensity. The bars shown represent the mean ± SE, and differences among groups were determined using one-way ANOVA. The asterisks indicate the significance of differences between the Vac-1 group and the indicated group. *: *p* < 0.05, **: *p* < 0.01.

Dendritic cells (DCs) play a very important role in connecting innate immunity and adaptive immunity and facilitating antitumor effects. The ability of DCs to capture antigens and their maturation rate are essential steps in immune activation. To further explore the mechanism of immune system activation mediated by our supramolecular protein chaperone, we extracted bone marrow-derived dendritic cells (BMDCs) and treated them with vaccines *in vitro*. The results obtained by confocal fluorescence microscopy (Figure 5A) indicated that most soluble RBITC-OVA was adhered to the cell membrane of the BMDCs at the 1 h time point, indicating poor uptake of RBITC-OVA by the DCs. However, RBITC-OVA loaded in Vac-1 or Vac-2 was largely absorbed by DCs and colocated with lysosomes at 1 h. The amount of RBITC-OVA taken up by the cells in the Vac-1 group was approximately 1.3 times that in the Vac-2 group (Figure 5B and 5C).

**Figure 6:**
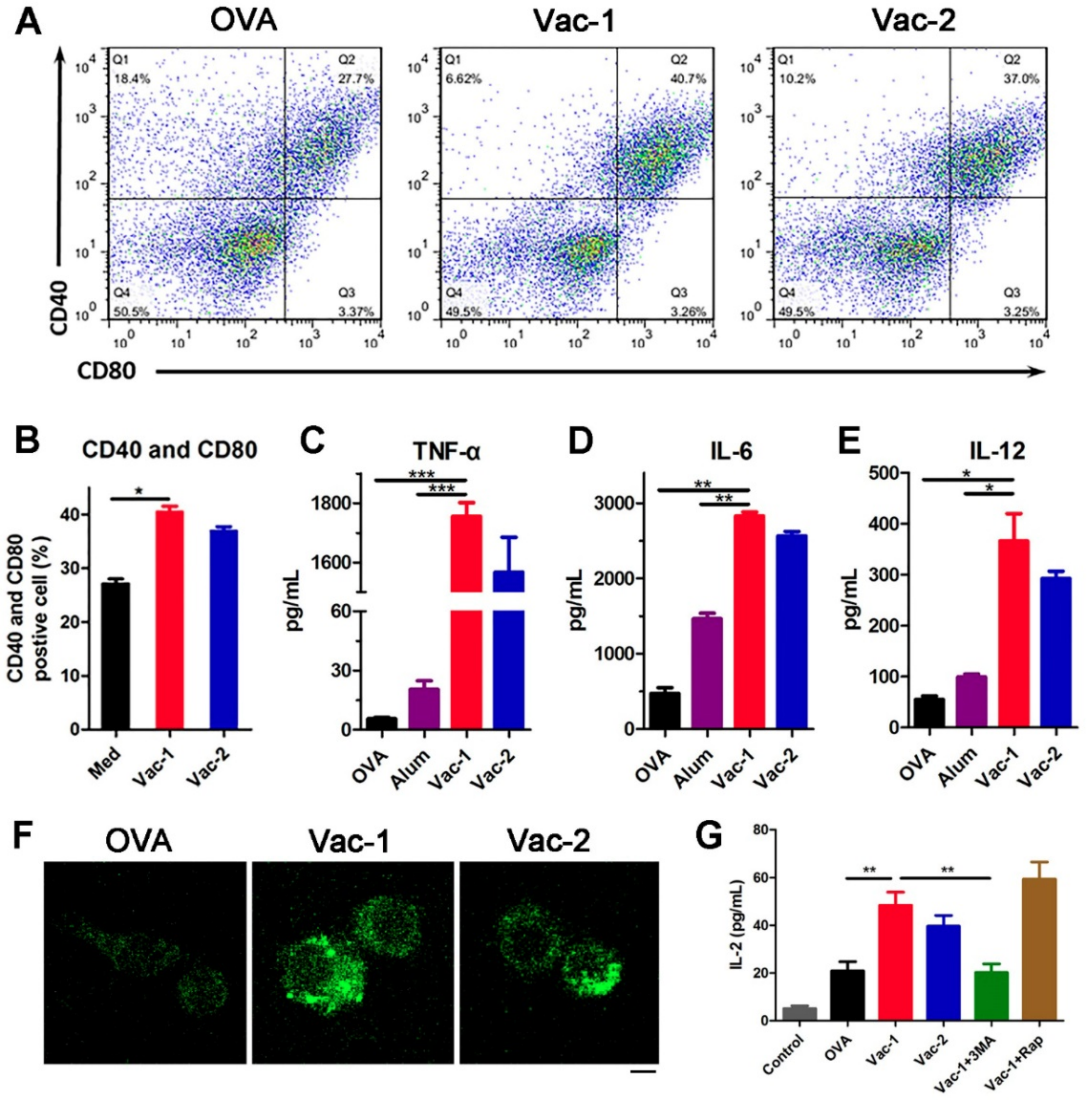
Vaccine promoting BMDCs maturation and Inflammatory cytokine release. (A, B) The expression levels of CD40 and CD80 in BMDCs were measured by flow cytometry. (C-E) The levels of TNF-α, IL-6 and IL-12 in cell culture supernatants were detected with ELISA kits. (F) LC3 puncta images of autophagosome in BMDMs stained with LC3-II antibody (FITC) were detected by a confocal microscope. Scale bar: 10 μm. (G) IL-2 cytokine in supernates secreted by BMDMs was measured by ELISA kits. The bars shown represent the mean ± SE, and differences among groups were determined using one-way ANOVA. The asterisks indicate the significance of differences between the Vac-1 group and the indicated group. *: *p* < 0.05, **: p < 0.01, and ***: p < 0. 001.

After capturing antigens, DCs will gradually mature and secrete a variety of cytokines and chemokines to stimulate other immune cells, ultimately promoting the immune response. After incubating DCs with different formulations for 24 hours, the supernatants were collected, and the DCs were incubated with PE-conjugated anti-CD40 and APC-conjugated anti-CD80 antibodies for another 30 minutes. The flow cytometric analysis results indicated that our supramolecular protein chaperone obviously promoted the maturation ratio of DCs to 40.7% (Figure 6A and 6B). Additionally, cytokine levels in the supernatant were determined by enzyme-linked immunosorbent assay (ELISA). The production of TNF-α, IL-6 and IL-12 in the Vac-1 group was higher than that in the Vac-2 group (Figure 6C-6E). In particular, the expression level of IL-12 in the Vac-1 group was 1.25 times that in the Vac-2 group. The high expression of IL-12 promoted the transformation of Th0 cells into Th1 cells, which is a precondition of cellular immunoreactions against a tumor.

### Vaccine recruiting autophagy for adjuvant function

Autophagy is a process of cellular self-cleaning and renewal. Autophagy in antigen-presenting cells (APCs) was recently demonstrated to be capable of enhancing the processing and presentation of antigens and inhibiting cancer development by regulating inflammation and immunity 44,45. To explore the influence of our hydrogel vaccines on autophagy pathway, we isolated the bone marrow-derived macrophages (BMDMs) from C57BL/6 mouse and treated them with different vaccines. Subsequently BMDMs were stained with LC3-II antibody to measure the level of microtubule associated protein 1 light chain 3B (LC3B)-II protein (a marker on autophagosomal membrane). LC3 fluorescent puncta of autophagosome in BMDMs were detected by laser confocal microscope. As the Figure 6F shown, BMDMs treated with Vac-1 formed more autophagosome than Vac-2, and none in untreated group, indicating Vac-1 effectively induced enhancement of autophgic actvities of macrophages. Besides, IL-2 level in BMDMs supernates was measured by ELISA kits. Compared to Vac-2 and soluble OVA, Vac-1 obviously enhanced the production of IL-2 (Figure 6G). When BMDMs pretreated with 3-methyl adenine (3MA, an inhibitor of autophagy), Vac-1 acquired a loss of IL-2 production. Nevertheless, when BMDMs stimulated with rapamycin (Rap, an inducer of autophagy), the production of IL-2 had a greater improvement in Vac-1 group. These results suggested our supramolecular protein chaperone can effectively recruit autophagy of APC for antigen processing and presentation, which contributed to enhancement of anti-tumor immunity.

### Vaccine promoting accumulation and retention of antigen in lymph nodes

**Figure 7:**
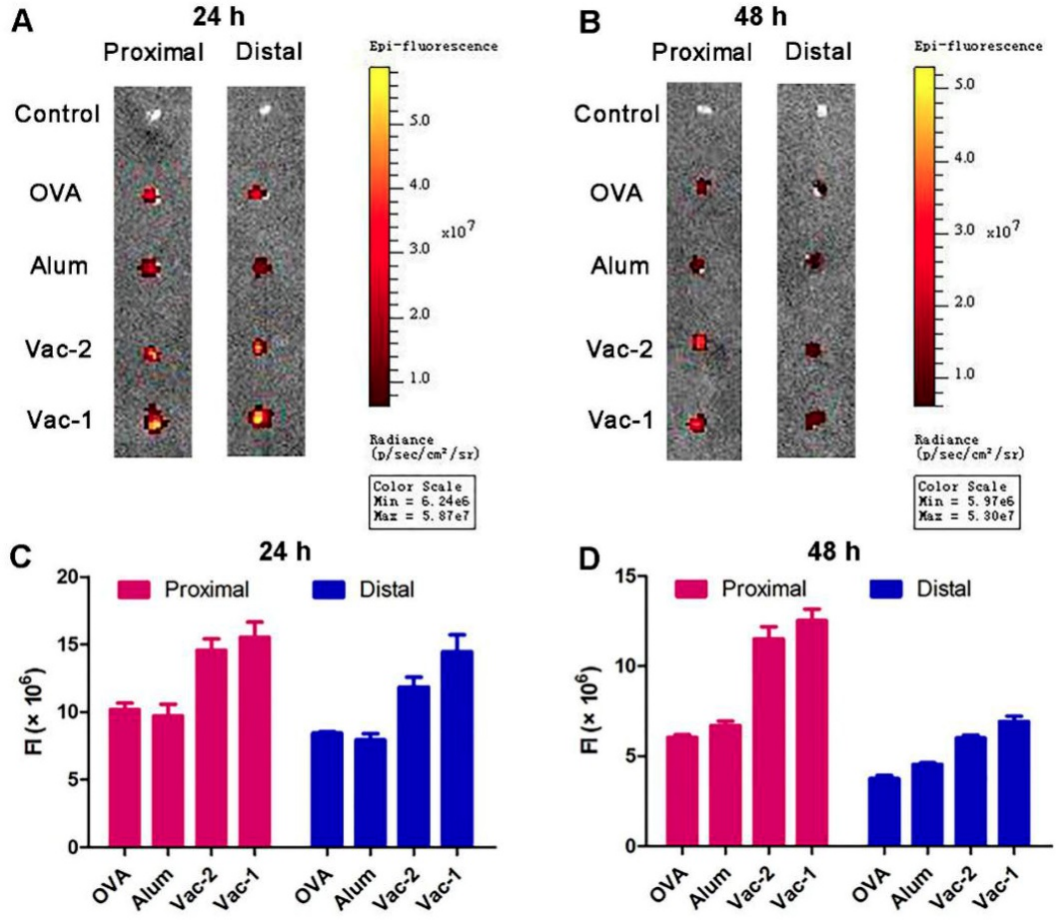
Vaccine promoting accumulation and retention of antigen in proximal and distal lymph nodes. (A-D) C57BL/6 mice were s.c. injected with soluble OVA-Rhod, L-gel formulated OVA-Rhod (L-gel/OVA), D-gel formulated OVA-Rhod (D-gel/OVA) or Alum encapsulated OVA-Rhod (Alum/OVA). The dose of OVA-Rhod, hydrogel, or Alum was 50, 500, and 1250 µg per mouse, respectively. After 24 h (A, C) or 48 h (B, D), the lymph nodes were obtained and analyzed using IVIS Lumina II.

The lymph nodes are important peripheral immune organs where lymphocytes receive antigen stimulation and produce an adaptive immune response. The accumulation and retention of antigens in lymph nodes are an important index to evaluate the efficacy of adjuvants. RBITC-labeled OVA in the Vac-1 and Vac-2 formulations was injected into the groin of C57BL/6 mice, and the proximal and distal lymph nodes were removed after 24 h and 48 h, respectively. Fluorescence intensity was measured *via* a small animal imaging system. As shown in Figure 7, compared to Vac-2, Vac-1 immensely enhanced antigen enrichment in both the proximal and distal lymph nodes at 24 h. The amount of antigen in the lymph nodes in the Vac-1 group was also higher than that in other groups at the 48 h time point. On the basis of the *in vivo* and *in vitro* results, our supramolecular protein chaperone effectively delayed the release of antigen, promoted the uptake of antigen and maturation of DCs, prolonged the retention time of antigens *in vivo*, and continuously stimulated the immune system.

### *In vivo* evaluation of therapeutical effect of vaccines

**Figure 8:**
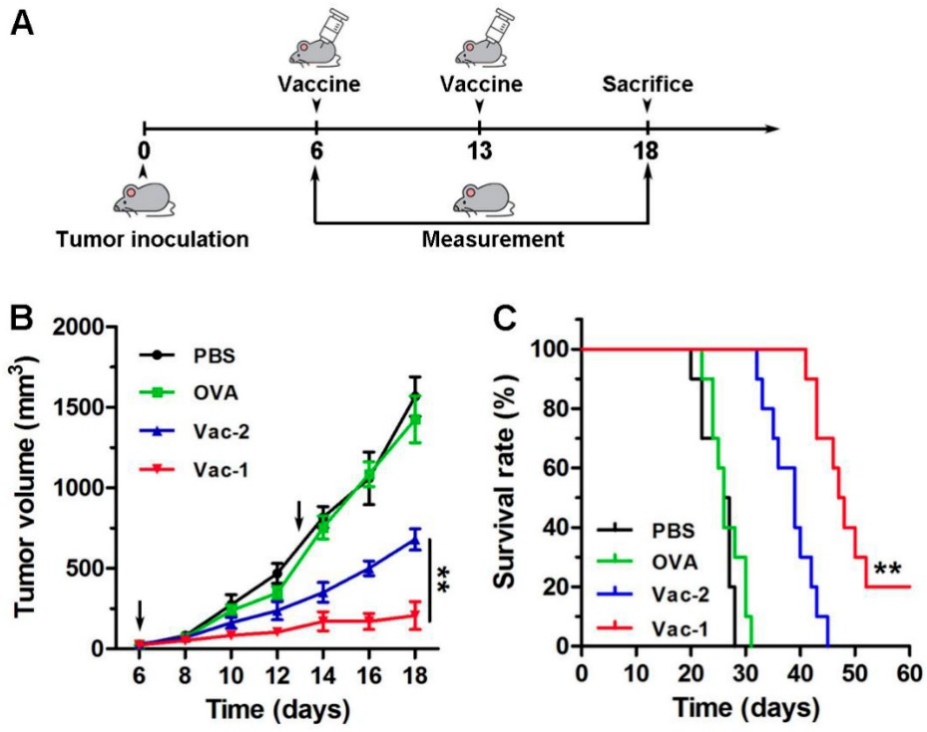
The evaluation of tumor therapeutic effect of vaccines. (A) The program of immunotherapy for E.G.7 tumor. (B) E.G.7 tumor-bearing C57BL/6 mice were vaccinated with PBS, OVA, Vac-1 or Vac-2, respectively. Tumor volume was monitored every 2 days. (C) The survival times of the mice vaccinated with PBS, OVA, Vac-1 or Vac-2.

Subsequently, a tumor inhibition assay was performed with E.G.7-OVA cells (Figure 8A). E.G.7-OVA cells were first inoculated onto the right buttock on day 0. The C57BL/6 mice received the first vaccination when the tumor volume reached approximately 50 mm^3^. The tumor size was recorded every two days. As shown in Figure 8B, compared to PBS, Vac-2 immensely inhibited tumor growth. However, Vac-1 had more powerful therapeutic efficacy than Vac-2 in inhibiting tumor growth. Moreover, Vac-1 immensely extended mouse survival (Figure 8B). No significant histopathological lesions were found in the major organs (Figure S11), confirming the good biocompatibility of Vac-1. These results clearly suggest the promise of applying our supramolecular protein chaperone in the development of vaccines to treat cancers.

**Figure 9:**
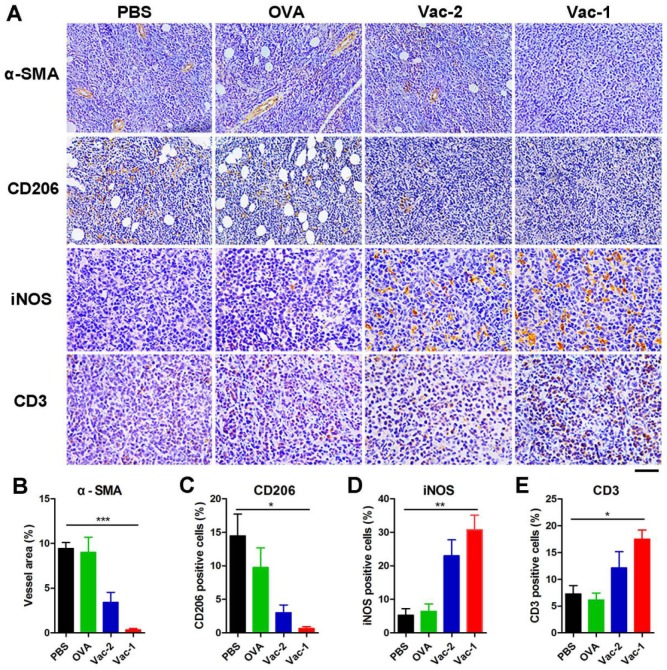
Immunohistochemical analysis of tumor tissues. (A) Representative immunohistochemical images of staining for α-SMA, CD206, iNOS and CD3 in HCC tissue microarray sections are shown (scale bar: 100 μm for α-SMA and CD206; 50 μm for iNOS and CD3). (B) The vessel area were calculated based on α-SMA antibody staining. (C) The M2-like macrophages were calculated based on CD206 antibody staining. (D) The M1-like macrophages were calculated based on iNOS antibody staining. (E) The infiltrating T lymphocytes were calculated based on CD3 antibody staining. Data presented as the mean ± SEM, n = 3. *p < 0.05, **p < 0.01, ***p < 0.001.

To further understand the antitumor mechanism of Vac-1, we analyzed the tumor microenvironment by immunohistochemistry (IHC). Tumor tissue samples from different groups were first incubated with an antibody against α-smooth muscle actin (α-SMA), which was a major component in smooth muscle cells in blood vessels. High expression of α-SMA represents an abundance of blood vessels, which is favorable for tumor invasion and metastasis. Our hydrogel vaccines, especially Vac-1, could tremendously inhibit the expression of α-SMA and tumor angiogenesis (Figure 9A and 9B). Subsequently, tissue samples were incubated with antibodies against CD206 (surface marker of M2-like macrophages) and iNOS (surface marker of M1-like macrophages), and the results indicated that Vac-1 promoted the production of M1 macrophages and inhibited M2 macrophages (Figure 9A, 9C and 9D). This M1 polarization was extremely beneficial for enhancing the antitumor immunoreaction 46-48. In addition, high expression of CD3 in the tumor tissue demonstrated that Vac-1 facilitated T lymphocyte infiltration into tumors (Figure 9A and 9E). Sufficient tumor-infiltrating lymphocyte (TIL) numbers are a precondition for the production of cytotoxic T lymphocytes, which are a key index for tumor immunotherapy.

To validate the therapeutic effect of the vaccines on other types of tumors, we established a B16-OVA tumor model by subcutaneously injecting B16-OVA tumor cells into the right buttock of C57BL/6 mice. The first vaccine inoculation was performed when the tumor volume reached 50 mm^3^ on day 6. The mice received another immunization on day 12. Over 14 days, compared to the PBS and OVA groups, both hydrogel vaccine groups, especially the Vac-1 group, exhibited effectively inhibited tumor growth, which limited the tumor volume to less than 280 mm^3^ and 780 mg (Figure S12A). The mice in the Vac-1 group had a higher survival rate than those in the other groups, and fifty percent of the mice survived for more than 50 days (Figure S12B). These results further demonstrated the potential of our protein chaperone as an extremely effective tumor vaccine adjuvant.

Our supramolecular protein chaperone was multifunctional in its anti-tumor effect. On one hand, our supramolecular protein chaperone exerted powerful anti-tumor immune activation by enhancing antigen present cells (APCs) function and inducing effector T lymphocytes/macrophages 49-52; On the other hand, the flurbiprofen-modified D-peptide reduced immune suppression by inhibiting COX-2/PGE_2_ pathway in tumor microenvironment 53-56. Our supramolecular protein chaperone had a great potential in the development of therapeutic tumor vaccines and other important vaccines 57.

## Conclusion

In summary, we developed a useful vaccine adjuvant for protein antigens. The supramolecular protein chaperone ***Comp. 1*** specifically interacted with various proteins with moderate binding affinities, followed by rapid folding into a β-sheet conformation and the formation of coassembled nanofibers/hydrogels with proteins. The supramolecular protein chaperone interacted with antigens by physical interactions, which facilitated both the cellular uptake of protein antigens and the release of the proteins after uptake. Therefore, the chaperone significantly stimulated specific antibody titers. In addition, the novel hydrogel vaccine showed great potential as a therapeutic tumor vaccine because of its high efficacy in stimulating specific cytotoxic T cells, inhibiting tumor angiogenesis, promoting M1 polarization of tumor-associated macrophages, and facilitating T lymphocyte infiltration. We showed the substantial potential of our supramolecular protein chaperone in protein delivery and vaccine development.
