# Supplementary material for: A supramolecular protein chaperone for vaccine delivery
Source: Theranostics. 2020 Jan 1;10(2):657–70. doi: 10.7150/thno.39132 (PMC6929975; doi:10.7150/thno.39132)
Supplement: Supplementary file 1 — Supplementary figures. [file thnov10p0657s1.pdf]

Supplementary information

A supramolecular protein chaperone for vaccine delivery

Zhongyan Wang, Yuna Shang, Zhaoqi Tan, Xiaoyan Li, Guoliang Li, Chunhua Ren*, Fuqiang Wang*, Zhimou Yang and Jianfeng Liu*

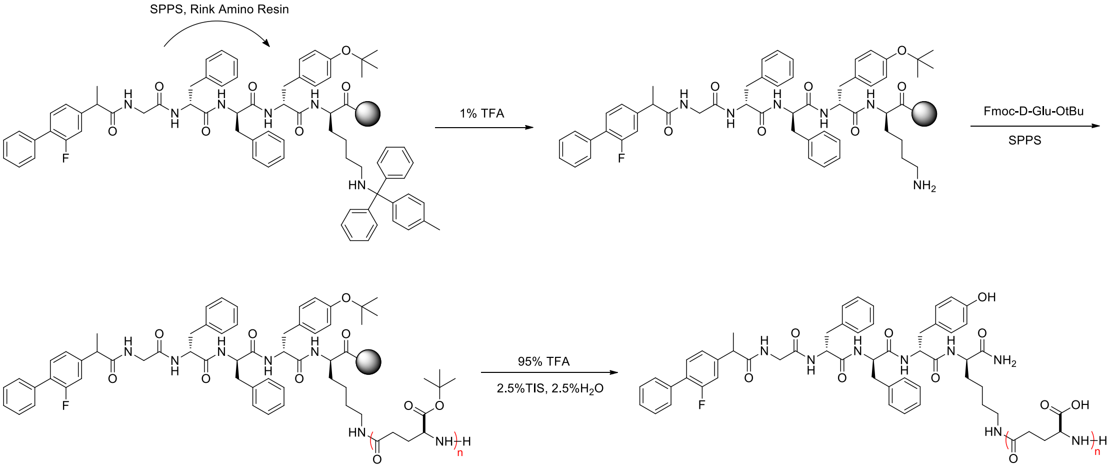

Scheme S1. Chemical structures and synthetic route of Fbp-G^{D}F^{D}F^{D}Y^{D}K(γE)$_n$-NH$_2$.

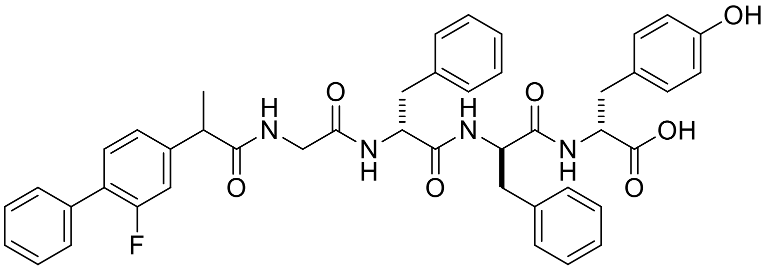

Scheme S2. The chemical structures of Fbp-G^{D}F^{D}F^{D}Y (***Comp. 2***).

Characteristic of compounds:

Fbp-G^{D}F^{D}F^{D}Y^{D}K(γE)$_2$-NH$_2$: ^{1}H NMR (400 MHz, DMSO) δ 8.39 – 8.16 (m, 1H), 8.09 – 7.99 (m, 1H), 7.90 (d, *J* = 7.6 Hz, 1H), 7.83 (s, 1H), 7.49 – 7.33 (m, 1H), 7.23 – 7.11 (m, 2H), 6.99 (s, 1H), 4.49 – 4.41 (m, 1H), 4.10 (d, *J* = 3.9 Hz, 1H), 3.87 (d, *J* = 5.7 Hz, 1H), 3.73 (d, *J* = 6.9 Hz, 1H), 3.63 – 3.47 (m, 1H), 3.09 – 2.87 (m, 1H), 2.46 (d, *J* = 1.8 Hz, 1H), 2.38 – 2.26 (m, 1H), 2.10 (t, *J* = 7.4 Hz, 1H), 2.02 – 1.90 (m, 1H), 1.30 (d, *J* = 7.0 Hz, 1H). M_{cal} =1144.2487, M_{exa} =1144.5145

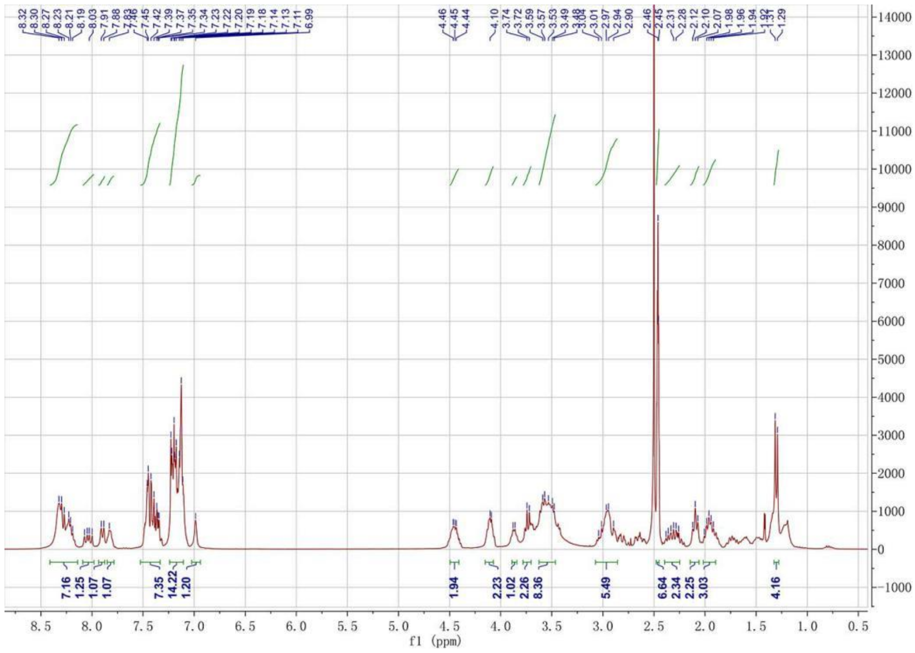

Fig. S1. ^{1}H NMR spectrum of Fbp-G^{D}F^{D}F^{D}Y^{D}K(γE)$_2$-NH$_2$.

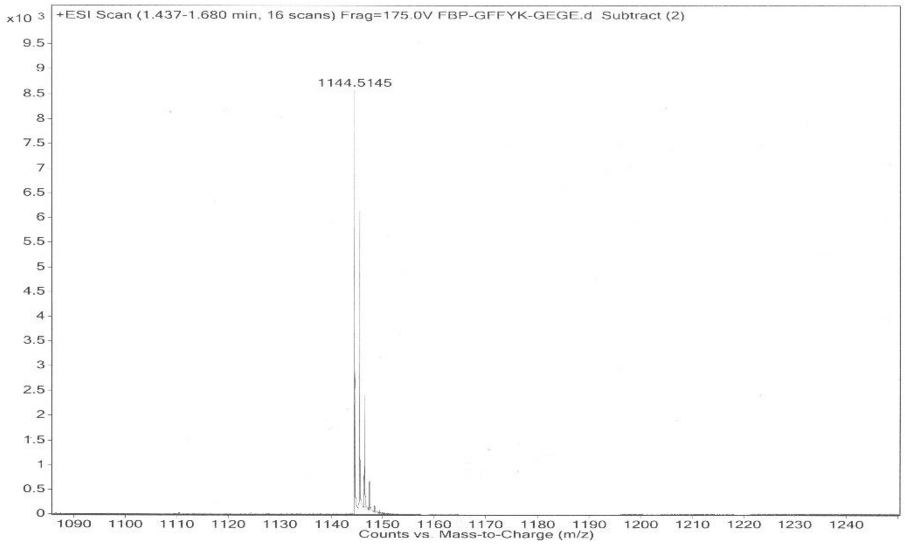

Fig. S2. HR-MS spectrum of Fbp-G^{D}F^{D}F^{D}Y^{D}K(γE)$_2$-NH$_2$.

Fbp-G^{D}F^{D}F^{D}Y: ^{1}H NMR (400 MHz, DMSO) δ 8.25 – 8.16 (m, 1H), 8.03 – 7.95 (m, 1H), 7.53 – 7.38 (m, 2H), 7.28 – 7.13 (m, 4H), 7.02 (d, *J* = 5.4 Hz, 1H), 6.66 (d, *J* = 5.8 Hz, 1H), 4.58 – 4.46 (m, 1H), 4.37 (d, *J* = 5.4 Hz, 1H), 3.78 – 3.72 (m, 1H), 3.65 – 3.54 (m, 1H), 3.48 (m, 1H), 3.05 – 2.91 (m, 1H), 2.87 – 2.71 (m, 1H), 2.69 – 2.59 (m, 1H), 1.37 – 1.28 (m, 1H). M_{cal} =758.83, $(M+H)^{+}$ =759.3189.

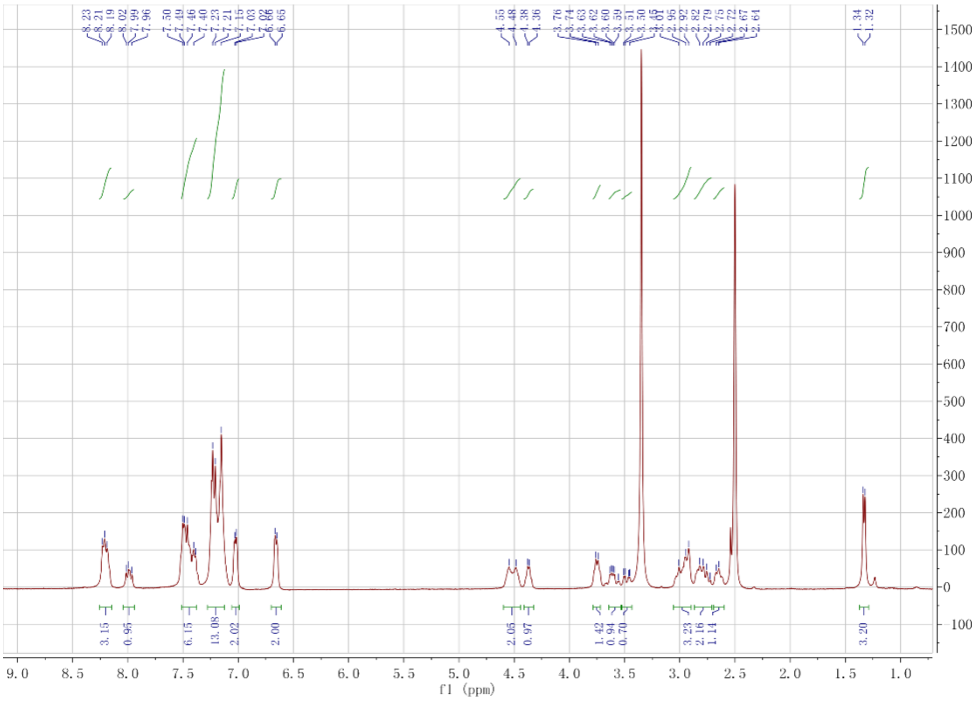

Fig. S3. ^{1}H NMR spectrum of Fbp-G^{D}F^{D}F^{D}Y.

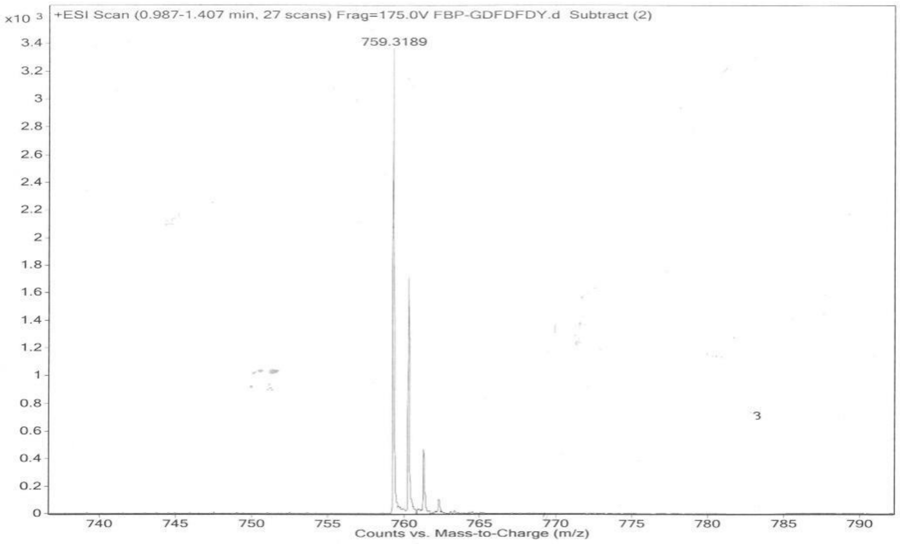

Fig. S4. HR-MS spectrum of Fbp-G^{D}F^{D}F^{D}Y.

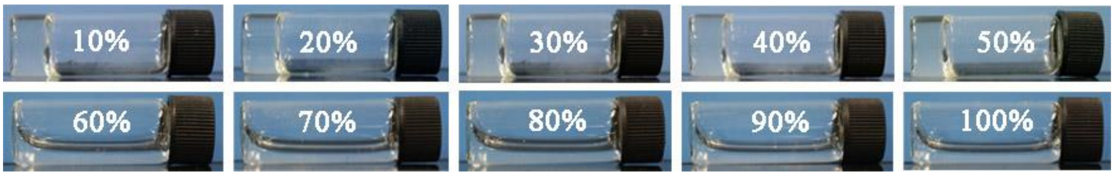

Fig. S5. Optical images of solution of ***Comp*. *1*** with the addition of different amounts of OVA.

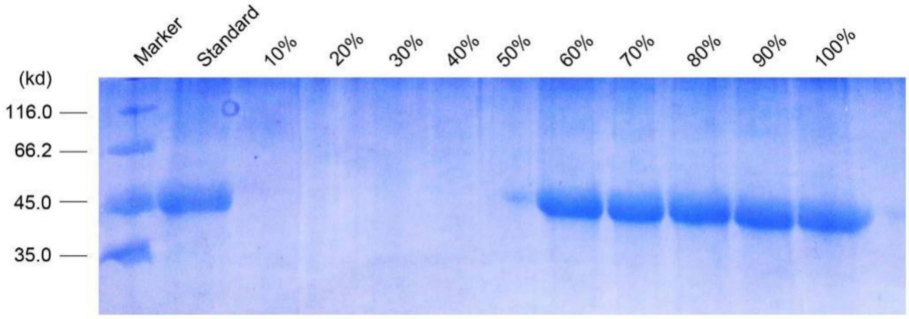

Fig. S6. SDS-PAGE image of ***Comp*. *1*** with OVA maximum loading rate.

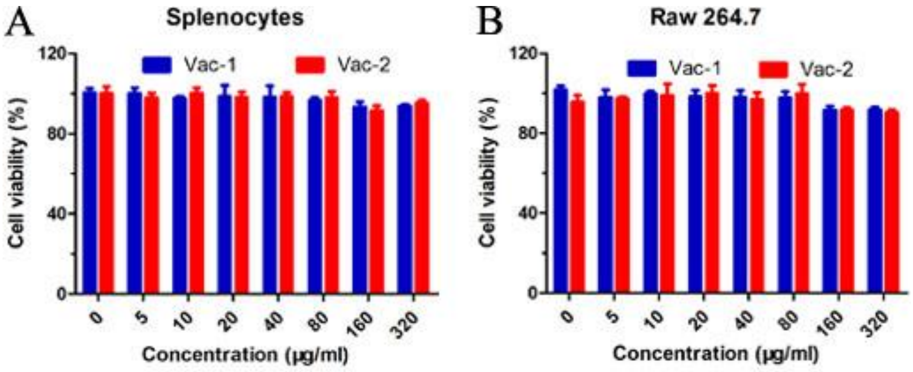

Fig. S7. The cytotoxicity effect of empty hydrogel on A) splenocytes and B) Raw 264.7 cells.

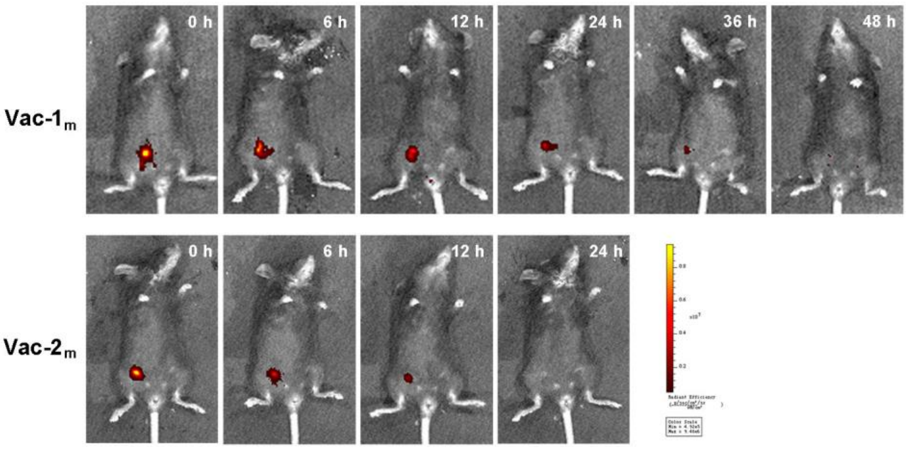

Fig. S8. The stability analysis in vivo of hydrogel vaccines

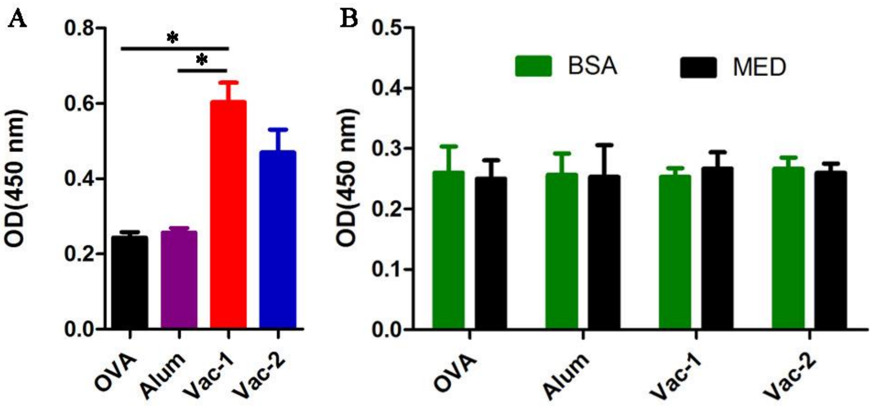

Fig. S9. The effect of Vac-1 and Vac-2 on inducing of antigen specific splenocytes proliferation *in vivo*.

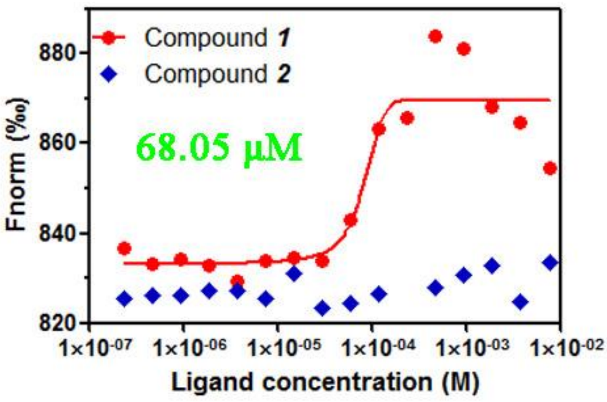

Fig. S10. The binding constants of compound ***1*** and ***2*** with HBsAg.

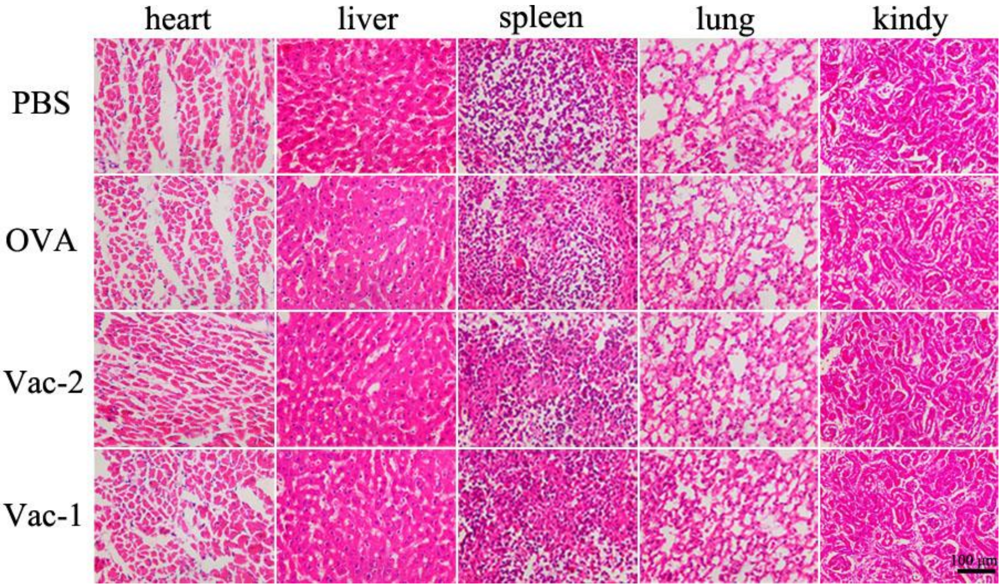

Fig. S11. Digital photograph of H&E staining of vital organ sections from PBS, OVA, Vac-2, or Vac-1 treated mice.

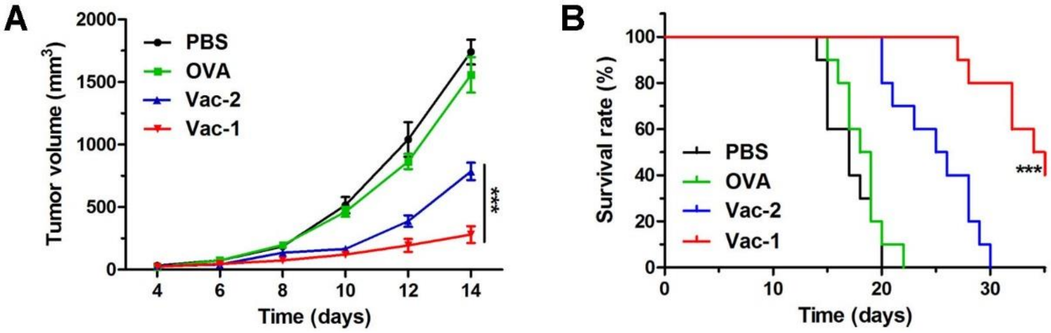

Fig. S12. A) B16-OVA tumor bearing C57BL/6 mice vaccinated with PBS, OVA, Vac-1, Vac-2. Mice were vaccinated and inoculated as described above, and tumor volume was monitored every 3 d. B) Survival time of mice vaccinated with PBS, OVA, Vac-1 and Vac-2, respectively.
